# Impellent impeller—Switching intra‐aortic balloon pump to IMPELLA‐CP after ST‐segment elevation myocardial infarction and refractory cardiogenic shock

**DOI:** 10.1002/ccr3.2261

**Published:** 2019-06-19

**Authors:** Marco Contarini, Francesco Costa, Maria Victoria Garcia Ruiz, Renato F. M. Scalise, Giovanni De Velli, Paola Murè, Paolo Mazzone, Giorgio Sacchetta, Giuseppe Andò

**Affiliations:** ^1^ Interventional Cardiology Unit Umberto I Hospital Syracuse Italy; ^2^ Department of Clinical and Experimental Medicine, Policlinico "Gaetano Martino" University of Messina Messina Italy; ^3^ UGC del Corazón, Servicio de Cardiología Hospital Clínico Universitario Virgen de la Victoria Málaga España

**Keywords:** cardiogenic shock, IMPELLA, intra‐aortic balloon pump, left ventricle assist device, ST‐segment elevation myocardial infarction

## Abstract

Cardiogenic shock is a dreadful complication of myocardial infarction. Despite widespread use and availability, intra‐aortic balloon pump (IABP) does not reduce mortality nor improves outcomes. Advanced mechanical circulatory support with IMPELLA system can substantially support hemodynamics and improve short‐term prognosis in patients with myocardial infarction and cardiogenic shock.

## 1 INTRODUCTION

Cardiogenic shock (CS) may complicate ST‐segment elevation myocardial infarction (STEMI) and is associated with a high fatality rate. Hemodynamic failure during CS represents a downward spiral in which myocardial ischemia drives to myocardial dysfunction, which, in turn, worsens myocardial and systemic ischemia. Intra‐aortic balloon pump (IABP) did not prove effective in this scenario^1^, ^2^ as more solid mechanical circulation support (MCS) devices are probably needed to revert the hemodynamic instability of CS. We describe the hemodynamic and metabolic changes of switching from IABP to a new percutaneous axial pump (ie, IMPELLA‐CP) in a patient with refractory cardiogenic shock after STEMI.

## 2 CASE REPORT

**Figure 1:**
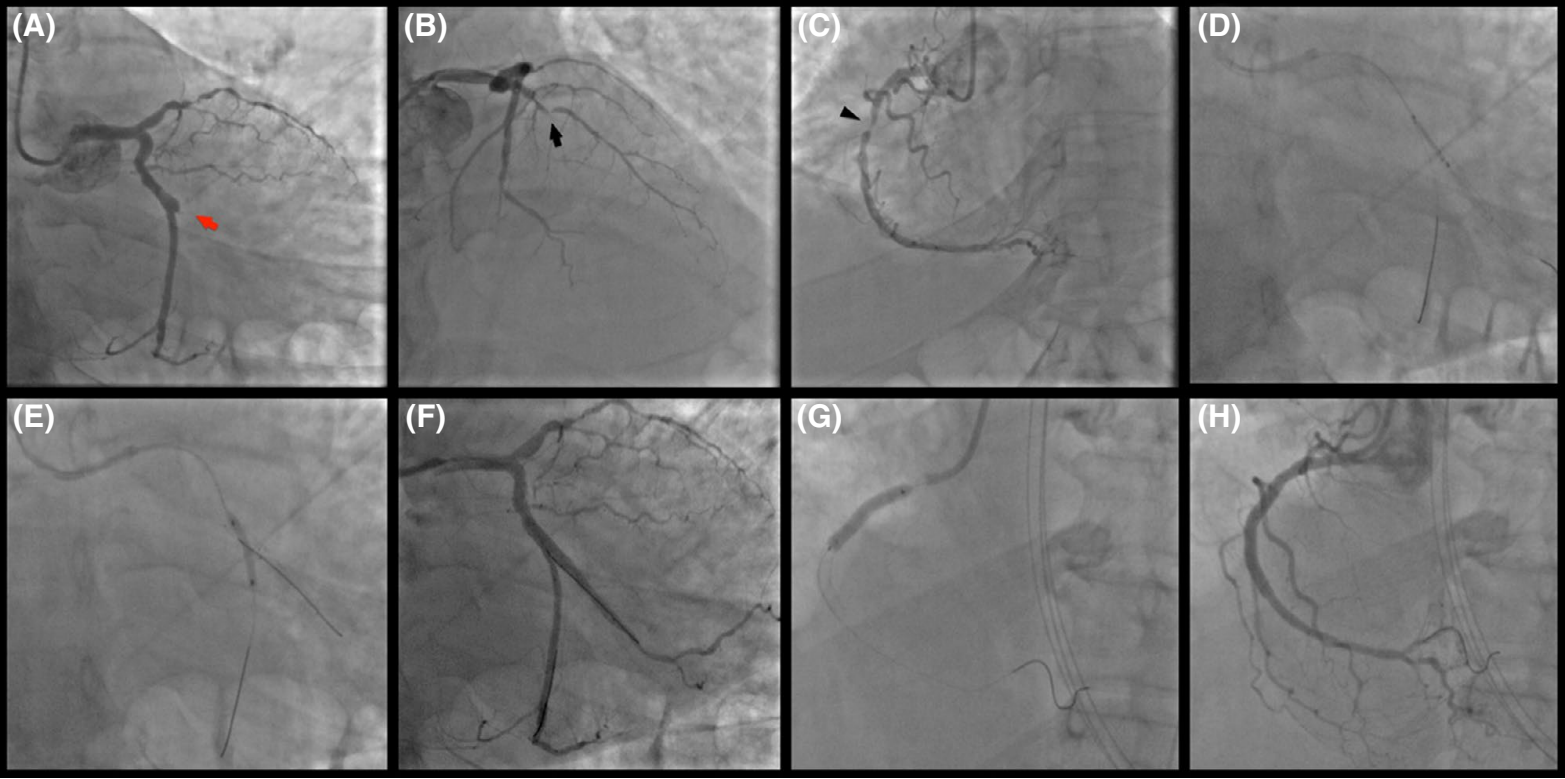
Primary Intervention: thrombotic occlusion of the first obtuse marginal (culprit lesion—red arrow) (A); chronic occlusion at the mid‐segment of the left anterior descending coronary artery (black arrow) (B); long lesion of the proximal right coronary artery (arrow‐head) (C). Two overlapping drug‐eluting stents in the main branch were used to treat the culprit at bifurcation (D) with provisional technique (E, F). Right coronary artery was treated with two overlapping drug‐eluting stents (G, H)

A 59‐year‐old man with no prior history of cardiovascular disease presented severely ill with oppressive chest pain during the last 6 hours and signs of inferolateral STEMI with cardiogenic shock. Reported cardiovascular risk factors were hypertension and diabetes mellitus. Emergent coronary angiography showed thrombotic occlusion of the first obtuse marginal, a chronic occlusion of the left anterior descending coronary artery and a long stenosis of the proximal right coronary artery. Revascularization of the marginal branch and the right coronary artery was performed, and IABP was placed without complications (Figure 1). After revascularization, blood pressure persisted low (80/60 mm Hg) despite escalating inotropic treatment, diuresis was reduced (<10 mL/h) and lactate levels increasing (>7 μmol/L), thus configuring a refractory cardiogenic shock (INTERMACS class 2).^2^ Symptoms of incipient pulmonary edema persisted despite diuretic treatment and ultrafiltration. Left ventricle function was poor (LVEF <20%). In light of the worsening conditions, we decided to remove the IABP and switch it with an IMPELLA‐CP (Abiomed Inc). The IMPELLA‐CP was placed percutaneously without complications, and correct positioning was confirmed by fluoroscopy and echocardiography. The perfusion parameters demonstrated a good pump function with a stable flow between 3.0 and 3.5 L/min.

**Figure 2:**
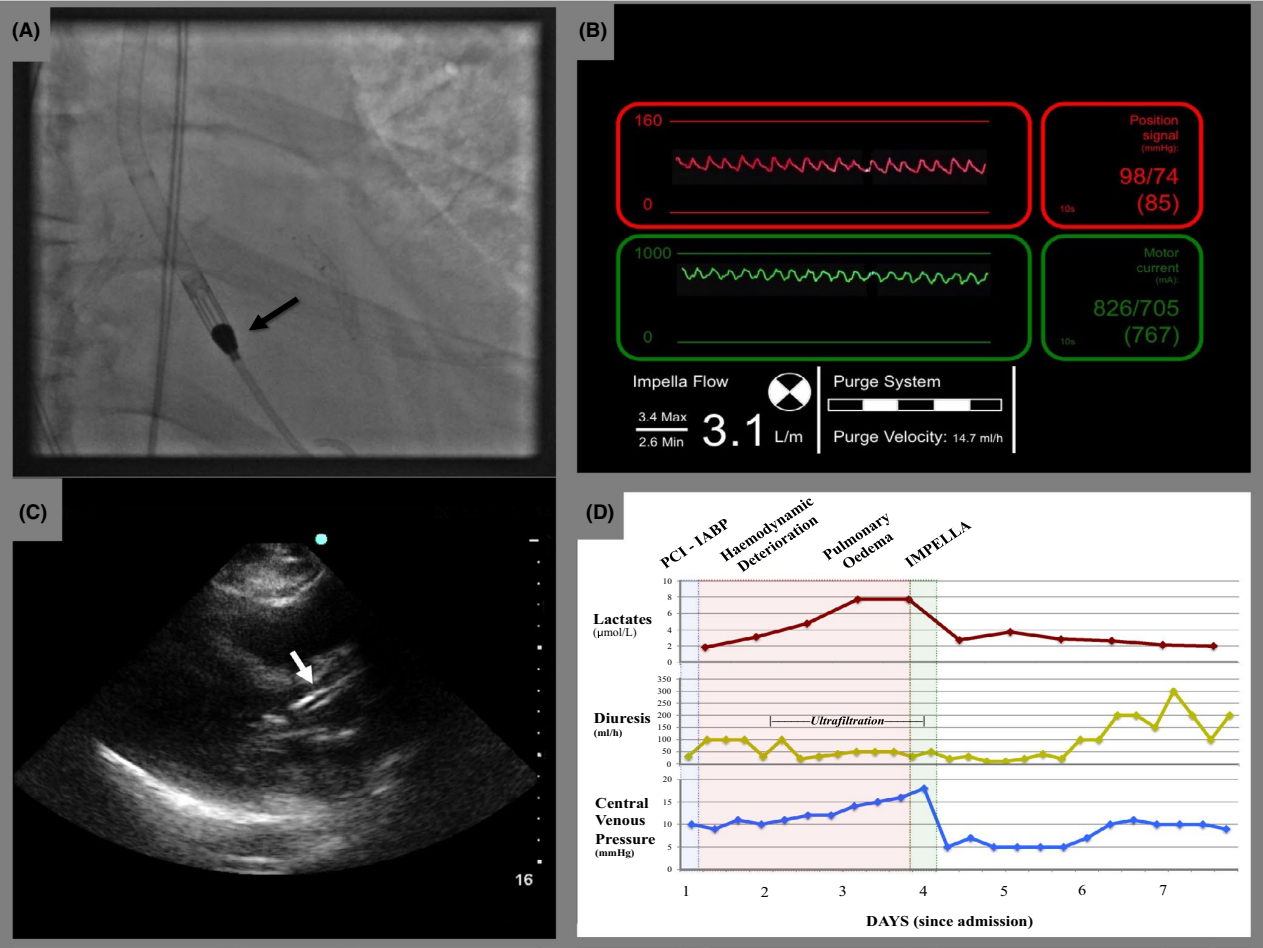
IMPELLA‐CP placement: IMPELLA‐CP was easily placed through the aortic valve and the pigtail end in proximity of the apex of the left ventricle (A). Good positioning and function were confirmed by satisfactory pressure waves at the device console (B) and by transthoracic echocardiography (C). Lactates, diuresis, and central venous pressure significantly improved after IMPELLA‐CP placement (D)

In the days after IMPELLA‐CP placement, we observed a progressive improvement of the hemodynamic and laboratory parameters (Figure 2). Lactate levels decreased, spontaneous diuresis recovered, and central venous pressure was maintained in the region of 10 mm Hg to guarantee an adequate preload. Left ventricle contractile function gradually improved (LVEF ≈ 30%) due to reduced afterload. Patient was progressively weaned from the device that was ultimately removed 10 days after implantation.

## 3 DISCUSSION

The present case highlights the metabolic and hemodynamic changes during the transition from a failing IABP to the IMPELLA‐CP system in the treatment of a refractory CS.

Cardiogenic shock complicating STEMI can persist despite optimal revascularization due to temporary myocardial stunning and is associated with an extremely high mortality risk.^3^, ^4^, ^5^ Since this condition might be reversible in days/weeks after the initial injury, an active hemodynamic support during this phase is required. IABP has been used for decades to treat cardiogenic shock; however, solid evidence from randomized clinical trials failed to demonstrate a benefit from its routine use.^1^ The use of more effective MCS devices, like the first‐generation IMPELLA which allows a flow up to 2.5 L/min, might intuitively improve the hemodynamic status in cardiogenic shock. However, IMPELLA and other MCS devices failed to improve short‐term survival in early studies of patients with CS.^3^

The new IMPELLA‐CP is an evolution of the first‐generation device and guarantees a maximal flow up to 4.0 L/min. This axial pump is inserted through a 14F introducer in the femoral artery and actively aspirates blood from an inlet cage in the left ventricle, expelling it in the ascending aorta. From a mechanistic standpoint, the main advantage of IMPELLA‐CP is the ability to unload the left ventricle reducing end‐diastolic pressure and myocardial oxygen demand, increasing cardiac output, coronary flow, and systemic perfusion. IMPELLA is more versatile as compared to other MCS devices, has a more favorable learning curve, and can be independently managed by the cath laboratory staff. This is particularly convenient in smaller centers and in remote areas that cannot rely on advanced perfusion systems, where such devices might be used as a bridge to hemodynamic stability or to allow transportation to a tertiary center.^4^ Nevertheless, it remains controversial whether IMPELLA‐CP may reduce mortality.^5^ Indeed, rigorous randomized clinical in this setting is extremely challenging to be carried out due to both the elevate complexity of the CS treatment and the ethical issues.

## 4 CONCLUSION

We report the hemodynamic and metabolic changes during the switch from IABP to IMPELLA‐CP in a patient with STEMI complicated by refractory CS. The new IMPELLA‐CP provided superior hemodynamic support as compared to IABP and may represent a good tool in the early management of CS. However, the clinical benefit of MCS devices to reduce mortality during CS has yet to be demonstrated.

## CONFLICT OF INTEREST

None declared.

## AUTHOR CONTRIBUTIONS

MC: performed the case, drafted, and revised critically the manuscript. FC: drafted and revised critically the manuscript. VGR: revised critically the manuscript. RS: revised critically the manuscript. GDV: revised critically the manuscript. PM: revised critically the manuscript. PM: revised critically the manuscript. GS: performed the case, drafted and revised critically the manuscript. GA: drafted and revised critically the manuscript.
